# A Diagnostic and Symptomatological Study on Trichomoniasis in Symptomatic Pregnant Women in Rafsanjan, South Central Iran in 2012-13

**Published:** 2015

**Authors:** Azita MANSHOORI, Sakineh MIRZAEI, Zarrintaj VALADKHANI, Mohammad KAZEMI ARABABADI, Mohsen REZAEIAN, Nahid ZAINODINI, Raza BAHRAMABADI, Mohammad ZARE-BIDAKI

**Affiliations:** 1 *Department of Gynecology and Obstetrics, School of Medicine, Rafsanjan University of Medical Sciences, Rafsanjan, Iran*; 2 *Department of Medical Parasitology, Pasteur Institute of Iran, Tehran, Iran*; 3 *Immunology of Infectious Diseases Research Center, Rafsanjan University of Medical Sciences, Rafsanjan, Iran*; 4 *Occupational Environment Research Center, Rafsanjan University of Medical Sciences, Rafsanjan, Iran*; 5 *Department of Microbiology, School of Medicine, Rafsanjan University of Medical Sciences, Rafsanjan, Iran*

**Keywords:** *Trichomonas vaginalis*, Diagnosis, Signs and symptoms, Iran

## Abstract

***Background:*** *Trichomonas vaginalis*, the causative agent of trichomoniasis, is responsible for more than half of all sexually transmitted infections (STIs). The present study aimed to determine the frequency of *T. vaginalis* infection and its clinical manifestations in symptomatic pregnant women in the area based on four different diagnostic methods.

***Methods:*** A total of 162 pregnant women with at least one sign or symptom of vaginosis, referred to two gynecologic and obstetrics clinics in Rafsanjan City, south central Iran, were randomly selected in 2012-13. Through speculum examination of patients by gynecologists, clinical diagnosis determined, vaginal discharge were collected by using two sterile cotton swabs from the posterior fornix and vagina pH was measured. Samples were examined by three diagnostic methods including wet mount, culture in TYI-S-33 medium and polymerase chain reaction (PCR).

***Results:*** *T. vaginalis* was detected in 19.5%, 27.2%, 56.2% and 51.6% of subjects according to diagnostic methods of clinical diagnosis, wet mount, culture and PCR, respectively. There was statistically significant relationship between *T. vaginalis* infection and patients' age, gestational age, marriage age, residence, educational level, parity. The symptomatological pattern in the 91 women infected with *T. vaginalis* was as follows: leukorrhea, 96.7%; urine frequency, 65.9%; odorous secretion, 63.3%; urogenital itching and irritation, 53.8%; vaginal inflammation, 47.3%; dyspareunia, 39.6%; and dysuria, 16.5%.

***Conclusion:*** Our results indicated a high prevalence of *T. vaginalis* in symptomatic pregnant women, very low sensitivity and relative high specificity of clinical diagnosis and wet mount technique compared to culture and PCR, as well as thatpregnancy increases the susceptibility to the infection in a gestational age-dependent manner.

## Introduction

According to WHO, about half a million new cases of curable sexually transmitted infections (STIs) including syphilis, gonorrhea, chlamydia and trichomoniasis occur annually throughout the word in adults aged 15-49 years.* Trichomonas vaginalis*, the causative agent of trichomoniasis, is responsible for more than half of all STIs, so that its new cases in world was reported as high as 276 million in 2008 (1) and additionally, it is the most common pathogenic protozoan of humans in the industrialized countries. The prevalence of the disease has been reported from 1% to 42% in various geographical regions of Iran (2). Symptoms and complications of symptomatic *T. vaginalis* infection in women include mild to severe urethritis, vulvovaginitis, cervicitis, preterm birth or low-birth-weight infant, cervical cancer and a higher susceptibility to HIV (3, 4). 

Clinical diagnosis of trichomoniasis in women is not a valid method since its signs and symptoms are very variable, and similar to other venereal diseases (4).

laboratory diagnosis of trichomoniasis through old method of microscopic examination of a wet-mount preparation of vaginal discharge or male urine sediment, because of the characteristic shape and motility of live *T. vaginalis*, has perfect specificity, but unfortunately it show a weak sensitivity as low as 50–70%, even in expert hands (4). Culture, using a variety of liquid and semi-solid media, is considered as the gold standard for diagnosis of trichomoniasis in women and use of specialized media increases the sensitivity to 85% to 95%. Therefore, wet mount followed by culture, merely on negative wet mounts, has recognized as the diagnostic method of choice for thrichomoniasis. However, the quality of microscopy examination on either wet mount or culture is strongly dependent on the skills and experience of the microscopist, as well as on the quality of the sample. Additionally, culture procedure is a relatively expensive and time-consuming method (4-6).

Recently, molecular tests based on nucleic acids and genetic markers of *T. vaginalis* has showed a higher diagnostic sensitivity compared to the combined approach i.e. wet mount followed by culture (5, 7). Briefly, progressive development and use of newer sensitive diagnostic tests are essential for the control of *T. vaginalis* infection as an important genitourinary pathogen in men and women (4). 

Based on our literature review, there is no investigation on the prevalence of trichomoniasis in the Rafsanjan County. Moreover, regarding the Kerman Province, we found only one report on the infection prevalence using direct smear and culture methods in Sirjan county in 1994 (8). 

The present study aimed to determine the frequency of *T. vaginalis* infection and its clinical manifestations in symptomatic pregnant women in the area based on four different diagnostic methods including clinical diagnosis, wet mount preparation, culture and polymerase chain reaction (PCR).

## Materials and Methods

***Study area***

The study was approved by institutional Research and Ethics Committee.

This study was performed in Rafsanjan City in the province of Kerman, in south central Iran, with a population of approximately 260,000, in more than 64,000 families. The city is at an altitude of approximately 1516 meters above sea level and located around 30° 24' north latitude and 55° 59' east longitude (9, 10).

***Study population and time***

From September 2012 to February 2013; 162 pregnant women with at least one sign or symptom of vaginosis, referred to two gynecologic and obstetrics clinics in Rafsanjan city, were randomly selected and enrolled in this study. These manifestations included vaginal discharge (leukorrhea), odorous secretion, urogenital itching and irritation, vagina inflammation, dysuria, urine frequency, dyspareunia. Patients’ information such as age, family income, occupation, educational status, Gestational age, number of past labors, abortion history, and the clinical signs were recorded in questionnaires.

***Sampling***

Through speculum examination of patients, vaginal discharges were collected by using two sterile cotton swabs from the posterior fornix. First swab transferred to a 15 ml screw-cap tube with sterile TYI-S-33 medium for culture method (11). The second swab kept in a sterile tube containing 1 ml normal saline for both wet mount microscopy and molecular detection of *T. vaginalis* by PCR. In addition, vagina pH was measured by moistening a pH paper with vaginal fluid obtained from the lateral vaginal wall.

***Microscopic examination***

The swab kept in normal saline was vigorously agitated and pressed against the side of the tube, then one drop of the fluid was directly mounted on a glass slide and examined microscopically at magnifications of ×100 and ×400 within 0.5-3 hours after sampling time.

Diamond’s TYI-S-33 medium (12) was used for culture of vaginal samples. Cultures were incubated at 37 ˚C for 7 days. At daily intervals, cultured samples were taken from tubes by disposable Pasteur pipettes in sterile condition and examined the same as described above, in the case of direct wet mounts, for motile *T. vaginalis*, pseudohyphae and/or budding yeast of *candida* and bacteria. 

***DNA extraction***

The tubes containing vaginal secretion in normal saline, were centrifuged at 2000 × g for 10 minutes. The supernatant was decanted and the pellet was suspended in 1 ml of sterile distilled water and then frozen at -20 ˚C until used, according to Lawing et al. (13) and Valadkhani et al. (5).

*T. vaginalis* DNA were extracted by using the CinnaPure DNAkit (CinnaGen Inc. Iran) according to manufacturer instructions and Valadkhani et al. (5). A volume of 400 μl of the Lysis buffer added to 100 μl of thawed sample and vortexed for 20 seconds. After adding 300 μl of precipitation solution, the sample was centrifuged at 12000 rpm for 1 min. The supernatant decanted and 400 µl of Wash buffer 1 added to the collection tube and centrifuged at 12000 rpm for 1 min. Wash buffer was completely decanted out and the added with Wash buffer 2 to collection tube and centrifuged at 12000 rpm for 1 min. In the next step, DNA pellet dissolved in 30 μl of sterile distilled water by gentle shaking and placing at 65 ˚C for 5 min. The unsolved material pelleted by spin for 1 min at 12000 rpm and finally, the supernatant containing purified DNA was used for PCR amplification.

***PCR*** ***protocol***

A pair of primers, targeting a specific and well-conserved region of β-tubulin (btub1) gene of *T. vaginalis* were designed, synthesized and used to amplify a DNA product of 220 bp. The gene encodes the amino acids of beta-tubulin protein, a main constituent of the parasite cytoskeleton (14). The primers sequences were as follows:

Forward: 5' ACT GGG CTA AGG GCT ACT ACA 3'; 

Reverse: 5' TTG GAG ATG GGA CGA TAG AG 3'.

PCR was performed according to the method described by Kazemi et al. (15) and Valadkhani et al. (5) with some modifications. The components of a total 20 μl volume of PCR reaction mixture consisted 10 μl Taq premix, 4 pmole each of the forward and reverse primers, 0.1 μg of template DNA and distilled water up to 20 μl.

The temperature profile consisted of an initial incubation at 94˚C for 5 min, and then 30 cycles, each of 30 sec at 94˚C for denaturation, 30 sec at 52˚C for annealing and 30 sec at 72 ˚C for extension, as well as a final elongation phase at 72 ˚C for 5 min. All PCR runs were performed using Flexcycler® thermocycler (Analytik Jena; Germany).

A volume of 4μl of amplified product was electrophoresed on a 1.5% agarose gel at 80 volts in Tris-Borate EDTA buffer. The size of DNA product was determined by commercial 100 bp weight markers (Cinnaclon, Iran).

***Statistical analysis***

The data entered into SPSS V18 and analyzed through descriptive and analytical statistics (Chi-Square, Mann-Whitney U, T Student, ANOVA and Kruskal-Wallis tests). A maximum significance level of 0.05 was assumed in all statistical tests.

## Results

***Diagnostic results***

Clinical diagnosis of 108 (out of 162) symptomatic pregnant women reported by gynecologist. The results of this diagnostic method which was based on their history and vaginal examinations were proportionally determined as: candidiasis 48.1%, bacterial vaginitis 16.7%, normal condition 11.1%, trichomoniasis 10.2%, mixed infection (simultaneous trichomonad and bacterial vaginitis) 9.3%, cervicitis 0.9% and vaginosis 3.7%.

The frequency and intensity of bacteria and fungi (yeasts and/or hyphae) observed in wet mounted and cultured samples are shown in Table 1.

Our study revealed that the frequency of *T. vaginalis* infection in symptomatic pregnant women varied between 19.5% up to a maximum of 56.2%, according to different diagnostic methods (Table 2). 

**Table 1:** The presence and intensity of bacteria and fungi in vaginal samples of symptomatic pregnant women according to the diagnostic methods (n=162)

**Method**	**Cell**	**Negative** **n (%)**	**Low** **n (%)**	**Moderate** **n (%)**	**High** **n (%)**
Wet mount	Bacteria	2 (1.6)	23 (20)	33 (26.4)	65 (52)
	Fungi	46 (38)	50 (41.3)	14 (11.6)	11(9.1)
Culture	Bacteria	12 (7.5)	68 (42.8)	38 (23.9)	41(25.8)
	Fungi	43 (26.9)	29 (18.1)	24 (15)	64 (40)

**Table 2:** The frequency of *Trichomonas vaginalis* in symptomatic pregnant women based on different diagnostic methods (n=162)

**Result** **Method**	***Negative*** **n (%)**	**positive** **n (%)**	**Total** **n**
Clinical diagnosis	87(80.5)	21(19.5)	108*
Wet mount	118(72.8)	44(27.2)	162
Culture	71(43.8)	91 (56.2)	162
PCR	76(48.4)	81(51.6)	157**

* Only 108 subjects out of 162, were clinically diagnosed by gynecologists

** 5 vaginal samples were not examined by PCR

The results in the four approach had statistically significant correlation with each other (*P* values ranged from 0.019 to 0.001), except clinical diagnosis against PCR results. Although the evaluation of three laboratory diagnostic tests were not methodologically designed in our study, but their sensitivity and specificity, with respect to different gold standard tests, were empirically calculated and shown in Table 3.

**Table 3:** Sensitivity and specificity of diagnostic techniques, with respect to different gold standard tests (all values are in percent)

** Gold standard **	**Clinical diagnosis**		**Wet mount**		**Culture**		**PCR**	
	**Sen.** *	**Spe.** *	**Sen.**	**Spe.**	**Sen.**	**Spe.**	**Sen.**	**Spe.**
Culture	27.9	97.1	65.6	96.4	100	100	63.3	66.1
PCR	22.4	85.1	46.2	76.4	73	55.4	100	100

* Sen. and Spe. are standing here for sensitivity and specificity, respectively

The vaginal pH ranged from 4 to 10 and mean pH in all patients was 6.2. Although the value was higher in women infected with *T. vaginalis* (6±1.32) and bacteria (5.95±1.31) than non-infected ones, but the difference was not statistically significant. However, vaginal pH in women harboring fungi was significantly lower than non-infected ones (5.78 vs. 6.24, *P*=0.05).

The frequency of *T. vaginalis* based on cultured method, was found to be significantly more common in rural women (*P*=0.007), in women with greater gestational ages (*P*=0.003), older ages (*P*=0.013) and higher educational levels (*P*=0.042), as well as in ones who got married later in life (*P*=0.071). There was no statistically significant relationship between *T. vaginalis* infection and patients' abortion history, parity, job and income.

***Symptomatological results***

**Fig. 1:**
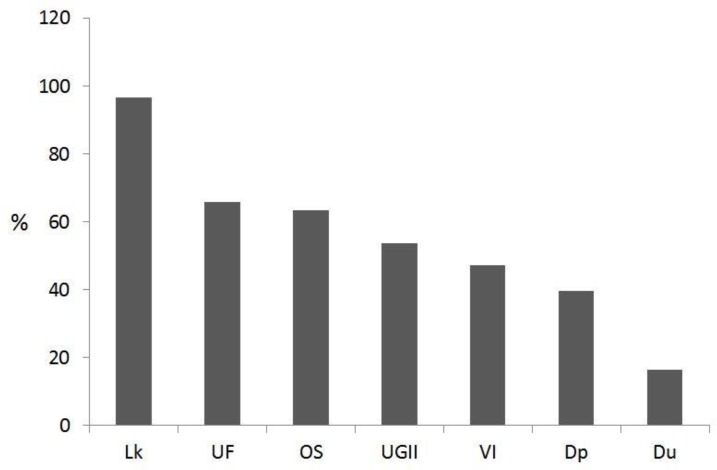
The percent of clinical manifestations observed in symptomatic pregnant women (n=162). Lk, leucorrhea; UF, urine frequency; OS, odorous secretion; UGII, urogenital itching and irritation; VI, vaginal inflammation; Dp, Dyspareunia; and Du, dysuria

All the patients have at least one of the known signs of trichomoniasis, but detailed clinical manifestations observed in our patients are shown in Fig. 1.

This symptomatological pattern was also seen in the 91 women infected with *T. vaginalis* based on the culture method as follows: leucorrhea, 96.7%; urine frequency, 65.9%; odorous secretion, 63.3%; urogenital itching and irritation, 53.8%; vaginal inflammation, 47.3%; Dyspareunia, 39.6%; and dysuria, 16.5%.

There was significant relationships between the presence of yeasts in vaginal smears and vaginal inflammation (*P*=0.022) as well as urogenital itching and irritation (*P*=0.015). Moreover, women with lower parity significantly more suffered from leukorrhea (*P*=0.039). 

Dyspareunia, urine frequency, dysuria, vaginal inflammation and urogenital itching and irritation were found to be significantly more common in women with lower vaginal pH (all with *P*<0.05).

## Discussion

While, based on physical examination and clinical diagnosis by gynecologists, a total of 19% (including all cases diagnosed as trichomonad or mixed infections) of our subjects had signs and symptoms of trichomoniasis. But the results of laboratory diagnostic methods of wet mount, culture and PCR revealed that 27%, %56.2 and 51.6% of symptomatic pregnant women were infected with *T. vaginalis*, respectively. This indicated the very low sensitivity of clinical diagnosis and wet mount technique (albeit both had relative high specificities) and the importance and necessity of sensitive methods for precise detection of the infection. Low sensitivity and specificity of our PCR results, compared to results of culture method as the gold standard test, may imply the need for optimizing the primers and technical procedures used in our study.

Few study is available that estimated the prevalence of trichomoniasis in Iranian symptomatic pregnant women. In a study on pregnant women in Semnan, northern Iran, using wet mount method, 5.5% cases were totally found to be infected with the parasite, but the prevalence in women at 16-20 and at 36 weeks of gestation were 29.9% and 70.1%, respectively (16). Additionally, in another study using wet mount technique in Hamadan, western Iran, while the prevalence of trichomonad infection in healthy women was reported 0.7%, but the parasite were detected as high as in 18.1% in symptomatic patients (17). These reports and our results is shown that pregnancy increases the susceptibility to the infection in a gestational age-dependent manner. 

In a study by Rasti et al., out of 450 pregnant women during delivery in Kashan, central Iran, only 2 (0.44%) cases found to be infected with *T. vaginalis*. Both of them had preterm birth, preterm premature rupture of membranes and low birth weight infants (18).

Nourian et al. in a study using wet mount and culture methods, detected *T. vaginalis *in 33 (3.3%) cases of 1000 pregnant women with gestational age ≥ 28 weeks, at the time of delivery in Zanjan, northwest Iran. They found a statistically significant relationship between *T. vaginalis* infection and preterm delivery as well as an increased risk of trichomoniasis in women with lower gestational age, higher age, higher parity and living in city (19).

In a study on 551 pregnant women in central Iran, *T. vaginalis* infection was diagnosed in 5.9%, 5% and 2% of subjects from Ardakan, Meybod and Yazd cities, respectively (20). As well, Mazloumi Gavgani et al. studied 1000 non-pregnant women in Tabriz, northwestern Iran revealed that 92 (9.2%) cases were positive for *T. vaginalis* based on culture method (21). In an investigation by Sharifi et al. on women in Sirjan, a city near and similar to our study area, 2.2% and 2.8% of subjects had *T. vaginalis* in wet mounted and cultured vaginal samples, respectively (8).

Studies on the epidemiology of trichomoniasis in pregnant women in various countries of the world have shown prevalence rates varying from 1.8% in Argentina (22), 2% in Nigeria (23), 7.7% in Brazil (24), 9.84% in Cuba (25), 10.2% in Congo (26), 19% and 21.3% in Papua New Guinea (27, 28), 32.2% in Zambia (29) and up to 46.9% in USA (30).

The widely varying results may be due to the differences in demographic and health conditions of study subjects and diagnostic methods used. These studies have reported a significant association between trichomoniasis and older age, HIV infection, prostitution, drug use, low birth weight (24, 26, 29, and 30) and not attending antenatal care (24).

Tibaldi et al. in a study using only wet mounts for detecting *T. vaginalis* in 27,172 Italian non-pregnant women, the rates of the infection in symptomatic and asymptomatic individual were 0.8% and 0.3%, respectively (31). Our higher rates may be resulted from pregnancy status of our subjects, as a predisposing factor, and use of more sensitive diagnostic methods, i.e. culture and PCR (31). The authors also showed that 49% of the symptomatic women had no microbiological etiology. Moreover, Wangnapi et al. reported that 59.1% of women with *T. vaginalis* infection were missed by clinicians (28). Our results were also consistent and indicated a low sensitivity of clinical diagnosis and urgent need for laboratory methods for accurate detection of the infection, even in symptomatic individuals.

Regarding the forth wave of AIDS in Iran, i.e. increasing the number of the HIV positive individuals due to sexual contacts, increased predisposition to HIV in *T. vaginalis* infected individuals and the trichomonad infection morbidity in pregnant women and its adverse effects on pregnancy outcome and fetal growth, it seems that the study on the epidemiology of *T. vaginalis* infection as a marker for other STIs and promoting diagnostic methods could help the health services to arrange programmes for better diagnosis, treatment and prevention of STIs and ultimately HIV infection in women, their partners and children.

## Conclusion

Our results indicated a high prevalence rate of *T. vaginalis* in the symptomatic pregnant women, very low sensitivity and relative high specificity of clinical diagnosis and wet mount technique, compared to culture and PCR methods, as well as an increasing effect of pregnancy on the susceptibility to the infection in a gestational age-dependent manner.
